# Impairment of Respiratory Chain under Nutrient Deficiency in Plants: Does it Play a Role in the Regulation of Iron and Sulfur Responsive Genes?

**DOI:** 10.3389/fpls.2015.01185

**Published:** 2016-01-05

**Authors:** Gianpiero Vigani, Jean-François Briat

**Affiliations:** ^1^Dipartimento di Scienze Agrarie e Ambientali-Produzione, Territorio Agroenergia, Università degli Studi di MilanoMilan, Italy; ^2^Biochimie and Physiologie Moléculaire des Plantes, Centre National de la Recherche Scientifique/Institut National de la Recherche Agronomique/SupAgro/UM2Montpellier, France

**Keywords:** iron, mitochondria dysfunctions, nutrient-responsive genes, respiratory chain, sulfur

## Abstract

Plant production and plant product quality strongly depend on the availability of mineral nutrients. Among them, sulfur (S) and iron (Fe) play a central role, as they are needed for many proteins of the respiratory chain. Plant mitochondria play essential bioenergetic and biosynthetic functions as well as they have an important role in signaling processes into the cell. Here, by comparing several transcriptomic data sets from plants impaired in their respiratory function with the genes regulated under Fe or S deficiencies obtained from other data sets, nutrient-responsive genes potentially regulated by hypothetical mitochondrial retrograde signaling pathway are evidenced. It leads us to hypothesize that plant mitochondria could be, therefore, required for regulating the expression of key genes involved both in Fe and S metabolisms.

## The interactions between Fe and S homeostasis in plants

Plant production and plant product quality strongly depend on the availability of mineral nutrients (Briat et al., 2015a,b). Among them, sulfur (S) and iron (Fe) play a central role, as they are needed for many proteins of essential metabolic processes. Indeed, Fe and S interact for the building of Fe–S clusters, which are essential prosthetic groups for photosynthesis, respiration, and many enzymatic reactions (Couturier et al., 2013). Fe and S interactions were documented both at physiological and molecular levels, although the mechanisms integrating the homeostasis of these two elements remain unknown. Leaf Fe concentration decreases in S-deficient tomato, which is consistent with the decrease activity of the root Fe uptake system in response to S starvation in tomato (Zuchi et al., 2009) and in *Arabidopsis* (Forieri et al., 2013). Reciprocally, Fe starvation modifies S homeostasis. In Fe-deficient *Arabidopsis* plants, S-metabolism-related genes (among which plasma membrane and tonoplast S transporters, and enzymes of the S assimilation pathway) are co-expressed with Fe-deficient genes (Schuler et al., 2011). For example, the *SULTR1;1* S transporter gene is down-regulated in the absence of Fe (Forieri et al., 2013). It was not confirmed by Paolacci et al. (2014), showing that most of the group 2 and 4, and some of the group 1, tomato S transporter genes are up-regulated under Fe deficiency. In *Graminaceous* plants, S starvation decreases mugineic acid (Fe[III]-chelators) synthesis (Kobayashi and Nishizawa, 2012) and release (Kuwajima and Kawai, 1997; Astolfi et al., 2006). In contrast, *YS1* (encoding a Fe[III]-mugineic acid transporter; Curie et al., 2009) gene expression increases in response to Fe deficiency (Astolfi et al., 2012). Fe deficiency-induced modifications of S metabolism were also investigated in durum wheat (Ciaffi et al., 2013). Genes encoding enzymes of the S assimilation pathway (APS reductase, ATP sulfurylase, sulfite reductase, and serine acetyltransferase), and activity of these enzymes, are up-regulated under Fe-deficient/S-sufficient conditions, as observed under S starvation conditions. However, not all the genes necessary for S assimilation are regulated in response to Fe scarcity. Some of them have their response to Fe or S deprivation uncoupled. For example, the *SULTR1.1* transporter gene expression is strongly induced in response to S deficiency but unaffected by Fe starvation (Ciaffi et al., 2013). Recently, tomato plants grown under both S and Fe deficiencies were shown to display an even more increased expression of sulfate transporters in shoot and root than those of plant grown under a single nutrient deprivation (Zuchi et al., 2015). Such synergistic effect of S and Fe deficiency interaction was observed also at the metabolic level where the content of some metabolites (i.e., asparagine, fumaric acid, and malic acid) changed in the roots of plants grown under both S and Fe deficiencies comparatively to single nutrient deprivation (Zuchi et al., 2015). The interactions between Fe and S metabolisms have been started to be described at a molecular level but no information is yet available about the mechanisms regulating these cross-talks. Modulation of the Fe–S cluster biogenesis and their relative abundance in response to various nutritional stresses has, however, been suggested to potentially regulate Fe–S interactions (Couturier et al., 2013; Forieri et al., 2013).

Mitochondrion is one of the cellular compartments playing a central role in the Fe–S interaction, since it is a site where Fe–S cluster assembly takes place.

For detailed information about Fe–S clusters, the readers can refer to two recent reviews given by Couturier et al. (2013) and Balk and Schaedler (2014). Fe–S clusters are prosthetic groups composed of Fe atoms and acid-labile inorganic sulfide. Generally, Fe atom's coordination with the protein backbone occurs via thiol groups of cysteinyl residues. The most common clusters found in plant proteins are [Fe_2_S_2_] and [Fe_4_S_4_] clusters. Their cellular biogenesis is not spontaneous. In both eukaryotes and prokaryotes, Fe–S clusters are inserted co- or post-translationally into apo-proteins through specific assembly machineries. It allows a correct folding or stability of the protein. Schematically, the assembly process can be divided into two steps. First, Fe–S clusters are built on scaffold proteins interacting with iron- and sulfur-delivery proteins. Second, carrier proteins transfer the preformed Fe–S clusters to target apo-proteins. The nature of the Fe donor is still a matter of debate. Sulfur comes from cysteine through the activity of cysteine desulfurases that are associated with specific proteins to be fully active. A persulfide (S^0^) is produced onto an active site cysteine. Since sulfur is always present in the S^2−^ oxidation state in Fe–S clusters, it explains why electrons are needed to reduce sulfane sulfur during the course of cluster assembly. A few additional proteins including ATP-hydrolyzing proteins or sulfur acceptors are also needed for this reaction. Fe–S cluster assembly machineries in plants are compartmented in three systems: the SUF (sulfur mobilization), ISC (iron–sulfur cluster), and CIA (cytosolic iron–sulfur cluster assembly) machineries for plastidial, mitochondrial, and cytosolic/nuclear Fe–S proteins, respectively. In addition, export machinery between mitochondria and the cytosol links the ISC and CIA machineries. The export and CIA machineries are specific to eukaryotes, whereas the SUF and/or ISC machineries are observed in most living organisms. Fe–S clusters perform a wide diversity of functions. It ranges from electron transfer to (de)hydration reactions, radical-generation, or disulfide cleavage. From biological point of view, the functionality of Fe–S proteins is required for sulfur and nitrogen assimilation, chlorophyll catabolism, DNA repair and replication, ribosome biogenesis, tRNA thio-modification, or co-enzyme (biotin, lipoic acid, and thiamine) synthesis.

## The impact of Fe and S deficiencies on mitochondrial respiration

Mitochondria contain numerous Fe–S cluster-containing proteins participating in the respiratory chain. Indeed, one single respiratory chain unit requires at least 10 different Fe–S clusters corresponding to ~30 Fe atoms and 30 S atoms (Couturier et al., 2013; Balk and Schaedler, 2014).

Among the mitochondrial metabolic processes, respiration is of central importance. Indeed, the respiratory rate is a key determinant of the growth decrease under a range of abiotic stresses (Atkin et al., 2005; Atkin and Macherel, 2009; Van Aken et al., 2009; Jacoby et al., 2011). Therefore, understanding the control and regulation of the respiratory processes is vital to improve the rate of plant growth and biomass production (Jacoby et al., 2012).

Recently, Schwarzländer et al. (2012) demonstrated that the respiratory chain dysfunctions affect the expression of a great number of genes, suggesting the importance of mitochondria in the regulation of gene expression by retrograde signaling pathways. Although the nature of the retrograde signal(s) was not yet identified, the mitochondrial transport of electrons has been considered as a potential upstream stimulus for the regulation of nuclear gene expression (Schwarzländer et al., 2012).

Considering the essential role of Fe and S for mitochondrial respiration, a deficiency of these nutrients affects the function of mitochondria.

Iron-deficient cucumber plant showed decreased root Fe content by about 80% with respect to the control plant. In these plants, mitochondrial function was strongly affected and the specific activity of each respiratory chain complexes decreased as follows: Complex I – 95%, complex II – 77%, complex III – 56%, complex IV – 50%, and complex V – 52%. At the same time, the external type II NAD(P)H dehydrogenase (ND_ex_) increased by about 100% in Fe-deficient plants (Vigani et al., 2009; Vigani and Zocchi, 2010). Similar changes were observed also in *Hyoscyamus albus* (Higa et al., 2010).

In S-deficient *Arabidopsis* plants, S content decreased by about 48% in leaf and 40% in root tissues. In these plants, the total mitochondrial respiration of complex I and complex IV capacities decreased, whereas the activity of ND_x_ increased. These changes were accompanied by a lower ATP level and a more reduced state of leaf and root cells. Particularly, in S-deficient *Arabidopsis* plants, complex I capacity decreased by 40 and 25% in leaf and root tissues, respectively, whereas Complex IV capacity decreased by about 37 and 30% in leaf and root, respectively. At the same time, ND_ex_ increased by 45 and 20% in leaf and root, respectively (Ostaszewska et al., 2014).

Interestingly, such functional changes of the respiratory pathway observed under Fe or S deficiencies were associated to changes occurring at the ultrastructural level (Ostaszewska et al., 2014; Vigani et al., 2015a). Indeed, both nutrient deficiencies lead to a lower mitochondrial matrix density as well as to a lower cristae number compared to the control mitochondria. Particularly, such morphological changes were quantified in Fe-deficient plants: the number of cristae per mitochondria decreased by 56% and the relative intracristae space decreased by 46% in Fe-deficient mitochondria compared to the control ones. Such impaired ultrastructure might reflect the metabolic status of mitochondria with a decreased respiration rate (Vigani et al., 2015a).

An important question aroused when taking into consideration that (i) respiratory chain dysfunctions might be a source of retrograde signaling pathways and that (ii) Fe and S deficiencies affect respiratory chain: Are mitochondria involved in the regulation of Fe- and S-responsive genes?

In yeast, mitochondria play an important role in the regulation of Fe-responsive genes (Ueta et al., 2012). Indeed, Fe homeostasis in *Saccharomyces cerevisae* is controlled primarily by the transcriptional activator Aft1p (Rutherford and Bird, 2004). The activation/inactivation of Aft1p relies on its interaction with extra-mitochondrial monothiol glutaredoxins Grx3p and Grx4p. The Aft1p–Grxps complex senses the status of the mitochondrial Fe–S cluster biogenesis, and in turn regulates *S. cerevisae* genes required for Fe uptake and storage. Indeed, during Fe starvation, Fe–S cluster assembly in mitochondria as well as dimeric Grx3/4p with bound Fe–S clusters is limited, thereby decreasing the interaction of Grx3/4p with Aft1p. Therefore, Aft1p is able to bind its target promoters in order to increase the expression level of the iron regulon. In contrast, in the presence of Fe, Grx3/4p binds Fe–S clusters that require functional mitochondria in order to be correctly assembled. As a result, the Grx3/4p–Fe–S complex interacts with Aft1p, leading to its disassociation from its target promoters and to a down-regulation of the iron regulon (Ueta et al., 2012). These findings suggest that, at least in yeast, mitochondria play a central role in the regulation of cellular Fe homeostasis. However, these mechanisms do not seem to occur in plants (Bernard et al., 2009; Knuesting et al., 2015).

Although very few data are available concerning mitochondria retrograde signaling pathway in plants (Schwarzländer and Finkemeier, 2013), an involvement of mitochondria in such a pathway has been suggested to regulate Fe and S homeostasis (Wirtz et al., 2012; Vigani et al., 2013).

Indeed, under Fe-deficiency conditions, activities of Fe-containing enzymes are down-regulated, and the corresponding metabolite pools could be consequently modified. Therefore, organelle retrograde signals could be produced from post-transcriptional and/or post-translational-mediated metabolic changes, and transduced for subsequent regulation of nuclear genes important for Fe uptake and homeostasis (Vigani et al., 2013). Recently, it has been observed that knocking down Mitochondrial Iron Transporter (MIT) reprograms the transcriptome and the metabolome of rice plants, suggesting that a local induction of Fe deficiency in mitochondrial compartment affects the expression of several nuclear genes (Vigani et al., 2015b). Considering S, the crucial step of its assimilation in plant cells is the synthesis of cysteine. Such reaction occurs in cytosol, plastid, and mitochondria, since O-acetylserine-thiol lyase (OASTL) is localized in all these three compartments (Takahaschi et al., 2011). Interestingly, the mitochondrial isoform of OASTL is likely involved in the sensing of the cysteine status, and in turn in the sensing of S status (Wirtz et al., 2012). Therefore, a mitochondrial retrograde feedback signal(s) has also been proposed for the S assimilatory pathway (Forieri et al., 2013).

## Genes differentially expressed in both Fe- and S-deficient plants and in mitochondrial-impaired plants

Considering the strong impact that Fe or S deficiencies have on mitochondrial functions, several biochemical processes related to this organelle might be a source to produce retrograde signals. In this context, the meta-analysis of transcriptomic data sets from *Arabidopsis* plants displaying mitochondrial dysfunction revealed interesting observations (Schwarzländer et al., 2012). This study considered 11 transcriptomic data sets from plants having either their mitochondrial functions genetically impaired or the mitochondrial respiratory chain inhibited with drugs at different points. The data sets from plants genetically impaired in their mitochondrial function corresponded to the following genetic backgrounds: *aox1a* (Giraud et al., 2008); *ndufs4* and *ndufa1* (Meyer et al., 2009); *msh1xrec*A (Shedge et al., 2010); *AP3:u-ATP9* and *AP9:u-ATP9* (Busi et al., 2011); *msd1-RNAi* and *prxII F* (Schwarzländer et al., 2012). On the other hand, some data sets were obtained from plants having their mitochondrial respiratory chain inhibited by the following chemical treatments: olygomicyn A and rotenone (Clifton et al., 2005), and Antimycin A (Schwarzländer et al., 2012).

Among the numerous genes affected in their expression in response to the various mitochondrial dysfunctions genetically or pharmacologically induced as mentioned above, many genes known to be regulated by Fe or S deficiencies can be observed.

Several genes exhibiting similar expression (up- or down-regulation) under Fe-deficiency (see list reported in Stein and Waters, 2012) conditions or under various mitochondrial dysfunction conditions were revealed. They are the transcription factor POPEYE (PYE, at3g47640); ZINC-INDUCED FACILITATOR (ZIF1, at5g13740); glutamate ammonia ligase (GLN1;4 at5g16570); a kelch repeat-containing protein (at3g07720); COPPER CHAPERONE (CCH, at3g56240); FERULIC ACID 5-HYDROXYLASE 1(FAH1, at4g36220); NICOTIANAMINE SYNTHASE 1 (NAS1, at5g04950); FERRITIN3 (FER3, at3g56090); BHLH039 (at3g56980); nodulin (at1g21140), and some genes encoding for unknown functions (at5g05250 and at3g56360) (Table 1). Furthermore, general mitochondrial dysfunctions affected other important Fe-responsive genes. They are BRUTUS (BTS at3g18290); FRO3 (at1g23020); OBP3-responsive gene (ORG1, at5g53450); OLIGOPEPTIDE TRANSPORTER (OPT3, at4g16370); S-ADENOSYLMETHIONINE SYNTHETASE (SAM1, at1g02500); METAL TOLERANCE PROTEIN A2 (MTPA2, at3g58810) (Table 1).

**Table 1:** **Genes differentially expressed under Fe deficiency (Stein and Waters, 2012) and under different mitochondrial impairments**.

**Accession**	**Description**		**Mitochondrial dysfunctions**									
			**complex I**			**complex III**		**AOX**	**ATP synthase**		**MnSOD**	**MGR**
		**−Fe**	**ROT**	***ndufs4***	***ndufs1***	**OLM**	***prxIIF + AA***	***aox1a***	***AP9:u-ATP9***	***AP3:u-ATP9***	***msd1-RNAi***	***msh1-recA***
at3g18290	EMB2454|EMB2454 protein binding/zinc ion binding BTS (BRUTUS)											
at5g13740	ZIF1|ZIF1 (ZINC INDUCED FACILITATOR 1)											
at1g18910	Protein binding/Zinc ion binding											
at1g23020	FRO3, ferric-chelate reductase											
at5g53450	ORG1 (OBP3-responsive gene 1);											
at3g47640	Basic helix-loop-helix (bHLH) family protein PYE (POPEYE)											
at4g16370	OPT3 (OLIGOPEPTIDE TRANSPORTER)											
at5g16570	GLN1;4; glutamate-ammonia ligase											
at1g02500	SAM1 (S-ADENOSYLMETHIONINE SYNTHETASE)											
at5g49760	Leucine-rich repeat family protein/protein kinase family protein											
at3g07720	Kelch repeat-containing protein											
at5g01600	FER1 ferric iron binding/iron ion binding											
at3g46900	COPT2; copper ion transmembrane transporter											
at4g30490	AFG1-like ATPase family protein											
at5g59520	ZIP2; transferase, transferring glycosyl groups/zinc ion transmembrane											
at5g67370	Unknown protein											
at3g56240	CCH (COPPER CHAPERONE); copper chaperone											
at4g36220	FAH1 (FERULIC ACID 5-HYDROXYLASE 1)											
at4g38950	Kinesin motor family protein											
at5g26820	ATIREG3 (IRON-REGULATED PROTEIN 3)											
at3g56360	Unknown protein											
at1g80830	NRAMP1											
at5g04950	NAS1 (NICOTIANAMINE SYNTHASE 1)											
at3g58810	|MTPA2 (METAL TOLERANCE PROTEIN A2)											
at3g09220	LAC7 (laccase 7)											
at2g29995	Unknown protein											
at1g01570	Fringe-related protein											
at5g36890	BGLU42 (BETA GLUCOSIDASE 42)											
at3g56090	ATFER3 (ferritin 3)											
at1g49000	Unknown protein											
at3g56980	BHLH039, ORG3|BHLH039; DNA binding/transcription factor											
at3g21240	4CL2 (4-COUMARATE:COA LIGASE 2)											
at5g05250	Unknown protein											
at5g47910	RBOHD (RESPIRATORY BURST OXIDASE HOMOLOG D)											
at1g21140	Nodulin, putative											

*The data sets from plants genetically impaired in their mitochondrial function corresponded to the following genetic backgrounds: aox1a, (loss of alternative oxidase); ndufs4 and ndufa1 (loss of complex I); msh1xrecA, (mitochondrial genome rearrangement, MGR); AP3:u-ATP9 and AP9:u-ATP9 (loss of mitochondrial ATP synthase); msd1-RNAi (loss of mitochondrial manganese superoxide dismutase, MSD1, protein) and prxII F (inhibition of complex III) (Curley et al., 2009). The data sets from plants having their mitochondrial respiratory chain inhibited by the following chemical treatments: olygomicyn A (OLM) and rotenone (ROT) (Bales and Perkeybile, 2012), and Antimycin A (AA) (Curley et al., 2009). Up-regulated genes are in green and down-regulated genes are in red*.

Among genes identified as similarly regulated under Fe deficiency and mitochondrial dysfunctions, *PYE* and *BTS* are of a particular interest. They characterize a transcriptional regulatory network to control Fe homeostasis in *Arabidopsis* (Long et al., 2010). PYE is a transcription factor necessary for the distribution of already imported Fe, whereas BTS, a functional RING E3 ubiquitin ligase, could be a post-translational regulator of the transcriptional regulatory network involved in the Fe-deficiency response (Selote et al., 2015). Among the potential targets of PYE identified in Long et al. (2010), 42 genes exhibited an expression level affected in the data sets from mitochondrial-impaired plants (Table 2). These observations underline the link between mitochondrial dysfunctions and the PYE/BTS regulatory system controlling Fe homeostasis. Interestingly, the expression of *PYE* and *BTS* is up-regulated when complex I is impaired. As Fe deficiency strongly affects complex I activity and protein synthesis (Vigani et al., 2009), mitochondria might be involved in the induction of such genes that are crucial for Fe homeostasis in plants.

**Table 2:** **PYE target genes (Long et al., 2010) affected by mitochondrial impairments (Schwarzländer et al., 2012)**.

**Accession**	**Description**	**Mitochondrial dysfunctions**									
		**complex I**			**complex III**		**AOX**	**ATP synthase**		**MnSOD**	**MGR**
		**ROT**	***ndufs4***	***ndufs1***	**OLM**	***prxIIF + AA***	***aox1a***	***AP9:u-ATP9***	***AP3:u-ATP9***	***msd1-RNAi***	***msh1-recA***
at5g13740	ZIF1 (ZINC INDUCED FACILITATOR 1111); antiporter 668 FORWARD										
at1g24400	LHT2 (LYSINE HISTIDINE TRANSPORTER 2)										
at1g23020	FRO3; ferric-chelate reductase										
at1g72440	EDA25 (embryo sac development arrest 25) 7273596 REVERSE										
at3g21640	FKBP42, calmodulin binding										
at5g14960	DEL2 (DP-E2F-LIKE 2); DNA binding/transcription factor										
at5g03210	Unknown protein										
at5g13730	SIG4 (SIGMA FACTOR 4); DNA binding										
at4g23010	UTR2|UDP-galactose transporter-related REVERSE										
at1g74790	Catalytic										
at3g13700	RNA-binding protein, putative										
at2g33710	AP2 domain-containing transcription factor family										
at5g24470	PRR5|APRR5 transcription regulator/two-component response regulator										
at3g02140	TMAC2 (TWO OR MORE ABRESGENE 2)										
at4g36920	AP2 (APETALA 2); transcription factor										
at5g04590	SIR; sulfite reductase (ferredoxin)/sulfite reductase										
at5g45410	Unknown protein										
at3g47650	Bundle-sheath defective protein 2 family/bsd2 family										
at5g14950	GMII (GOLGI ALPHA-MANNOSIDASE II);										
at1g72460	Leucine-rich repeat transmembrane protein kinase										
at4g00585	Unknown protein										
at1g56220	Dormancy/Auxin associated family protein										
at1g03090	Symbols: MCCA|MCCA; methylcrotonoyl-CoA carboxylase										
at2g46930	Pectinacetylesterase, putative										
at2g30090	GCN5-related N-acetyltransferase (GNAT) family protein										
at1g68580	Agent domain-containing protein/Bromo-adjacent homology (BAH)										
at1g23030	Armadillo/Beta-catenin repeat family protein containing protein										
at2g46920	POL (poltergeist); protein serine/threonine phosphatase										
at3g47420	Glycerol-3-phosphate transporter, putative										
at3g55430	Glycosyl hydrolase family 17 protein										
at3g15200	Pentatricopeptide (PPR) repeat-containing protein										
at5g03140	Lectin protein kinase family protein										
at3g47430	PEX11B										
at4g01730	Zinc ion binding										
at1g11840	ATGLX1 (GLYOXALASE I HOMOLOG);										
at1g56430	NAS4 (NICOTIANAMINE SYNTHASE 4);										
at3g57070	Glutaredoxin family protein										
at3g15210	ERF4									
at5g45430	Protein kinase, putative										
at2g24550	Unknown protein										
at5g03230	Unknown protein										
at5g45310	Unknown protein										

*The data sets from plants genetically impaired in their mitochondrial function corresponded to the following genetic backgrounds: aox1a, (loss of alternative oxidase); ndufs4 and ndufa1 (loss of complex I); msh1xrecA, (mitochondrial genome rearrangement, MGR); AP3:u-ATP9 and AP9:u-ATP9 (loss of mitochondrial ATP synthase); msd1-RNAi (loss of mitochondrial manganese superoxide dismutase, MSD1, protein) and prxII F (inhibition of complex III) (Curley et al., 2009). The data sets from plants having their mitochondrial respiratory chain inhibited by the following chemical treatments: olygomicyn A (OLM) and rotenone (ROT) (Bales and Perkeybile, 2012), and Antimycin A (AA) (Curley et al., 2009). Up-regulated genes are in green and down-regulated genes are in red*.

Similar to what was observed for Fe, several S-responsive genes (Maruyama-Nakashita et al., 2006) were also strongly up-regulated under mitochondrial dysfunctions. These genes are encoding for S transporters SULTR1;2 (at1g78000), SULTR3;4 (at3g15990), SULTR4;2 (at3g12520), and other crucial S-responsive genes such as LSU1 (at3g49580), BGLU28 (at2g44460), and SDI1 (at5g48850) (Table 3).

**Table 3:** **Genes differentially expressed under S deficiency (Maruyama-Nakashita et al., 2006) and under different mitochondrial impairments**.

**Accession**	**Description**		**Mitochondrial dysfunctions**									
			**complex I**			**complex III**		**AOX**	**ATP synthase**		**MnSOD**	**MGR**
		**−S**	**ROT**	***ndufs4***	***ndufs1***	**OLM**	***prxIIF + AA***	***aox1a***	***AP9:u-ATP9***	***AP3:u-ATP9***	***msd1-RNAi***	***msh1-recA***
at5g23050	AAE17 (ACYL-ACTIVATING ENZYME 17											
at1g12200	Flavin-containing monooxygenase family protein|											
at3g05400	Sugar transporter, putative											
at1g08920	Sugar transporter, putative											
at2g22330	CYP79B3; electron carrier/heme binding/iron ion binding											
at1g78000	SULTR1;2 (SULFATE TRANSPORTER 1;2											
at3g15990	SULTR3;4 (SULFATE TRANSPORTER 3;4)											
at1g36370	SHM7 (serine hydroxymethyltransferase 7)											
at2g44460	BGLU28 (BETA GLUCOSIDASE 28)											
at5g40670	PQ-loop repeat family protein/transmembrane family protein											
at3g49580	LSU1 (RESPONSE TO LOW SULFUR 1)											
at5g26220	ChaC-like family protein											
at5g48180	NSP5 (NITRILE SPECIFIER PROTEIN 5)											
at3g47960	Proton-dependent oligopeptide transport (POT) family protein											
at1g64170	ATCHX16 (CATION/H+ EXCHANGER 16											
at5g48850	ATSDI1 (SULFUR DEFICIENCY-INDUCED 1)											
at3g12520	SULTR4;2; sulfate transmembrane transporter											
at1g04770	Male sterility MS5 family protein											
at1g75280	Isoflavone reductase, putative											
at4g13430	IIL1 (ISOPROPYL MALATE ISOMERASE LARGE SUBUNIT 1)											
at3g56040	UGP3 (UDP-GLUCOSE PYROPHOSPHORYLASE 3)											
at5g37980	NADP-dependent oxidoreductase, putative											
at1g78370	ATGSTU20 (GLUTATHIONE S-TRANSFERASE TAU 20);											
at5g43780	APS4; sulfate adenylyltransferase (ATP)											
at4g25100	FSD1 (Fe SUPEROXIDE DISMUTASE 1)											
at4g39950	CYP79B2; electron carrier/heme binding/iron ion binding monooxygenase/oxygen binding											
at5g23020	IMS2 (2-ISOPROPYLMALATE SYNTHASE 2)											
at4g01430	Nodulin MtN21 family protein											
at4g31500	CYP83B1 (CYTOCHROME P450 MONOOXYGENASE 83B1)											
at5g23010	MAM1 (METHYLTHIOALKYLMALATE SYNTHASE 1											

*The data sets from plants genetically impaired in their mitochondrial function corresponded to the following genetic backgrounds: aox1a, (loss of alternative oxidase); ndufs4 and ndufa1 (loss of complex I); msh1xrecA, (mitochondrial genome rearrangement, MGR); AP3:u-ATP9 and AP9:u-ATP9 (loss of mitochondrial ATP synthase); msd1-RNAi (loss of mitochondrial manganese superoxide dismutase, MSD1, protein) and prxII F (inhibition of complex III) (Curley et al., 2009). The data sets from plants having their mitochondrial respiratory chain inhibited by the following chemical treatments: olygomicyn A (OLM) and rotenone (ROT) (Bales and Perkeybile, 2012), and Antimycin A (AA) (Curley et al., 2009). Up-regulated genes are in green and down-regulated genes are in red*.

SULTR1;2 is a high affinity ${\text{SO}}_{4}^{2-}$ transporter from *Arabidopsis* (group 1), which mediates sulfate uptake into roots (Gigolashvili and Kopriva, 2014). SULTR 3;4 is a member of group 3 of S-tranporters, which are likely to be involved in ${\text{SO}}_{4}^{2-}$ translocation from root to shoot, whereas SULTR4;2 (group 4) functions in vacuolar export of ${\text{SO}}_{4}^{2-}$.

BGLU28 and SDI1 have received considerable attention. BGLU28 is the most strongly up-regulated gene characterized in several of the studies related to S deficiency. It encodes a protein hypothesized to act by releasing S from glucosinolates, a major potential S storage compound in the vacuole (Maruyama-Nakashita et al., 2003, 2006; Dan et al., 2007). SDI1 is annotated as a protein similar to male sterility family protein MS5, and recent evidences suggested that its expression level could act as a biosensor of S nutrient status (Howarth et al., 2009).

Interestingly, these S-responsive genes are strongly up-regulated under a specific mitochondrial impairment: rotenone treatment, which inhibits complex I activity (Schwarzländer et al., 2012). As recently reported, S deficiency affects complex I activity (Ostaszewska et al., 2014) likely because this complex has a high need of Fe–S cluster-containing proteins. Therefore, an inhibition of complex I might trigger the expression of S-responsive genes. Interestingly, such genes are crucial for S-sensing and signaling. Indeed SULTR1;2 has a major role in ${\text{SO}}_{4}^{2-}$ transport, and it has been recently suggested to act as a possible S-transceptor (Zheng et al., 2014). Furthermore, the transcriptional regulation of the *SULTR1;2, BGLU28*, and *SDI1* genes is under the control of SLIM1, a central transcription factor regulating the S-response in plants. Therefore, under S deficiency, the affected complex I activity might participate in the regulation of such genes through an unknown retrograde signaling pathway.

## Conclusion and perspective

**Figure 1:**
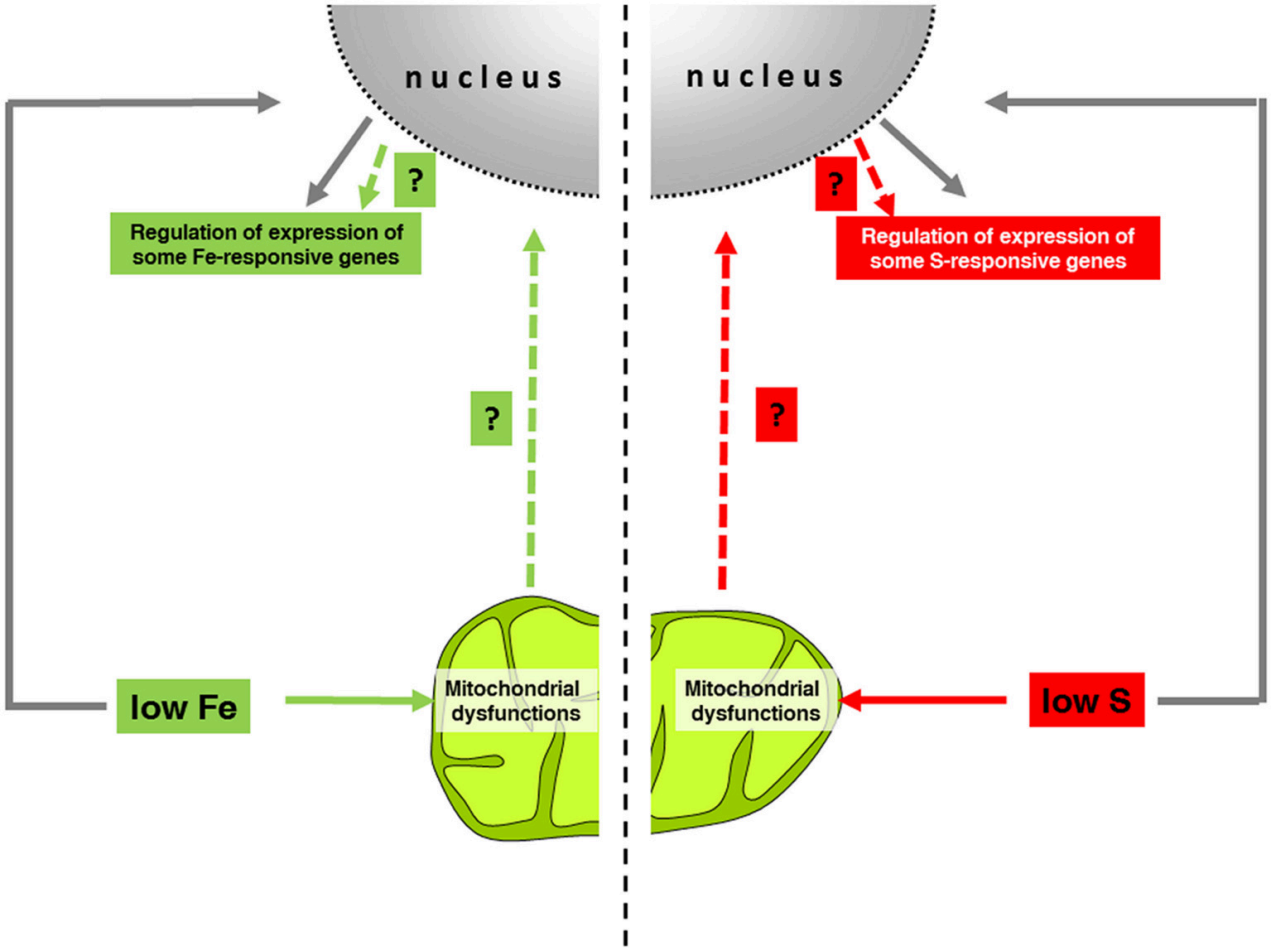
**Schematized model of the possible role of mitochondria in the regulation of nutrient-responsive genes**. As Fe and S are essential elements for Fe–S cluster-containing proteins, deficiencies of such nutrients trigger mitochondrial dysfunctions mainly at the respiratory chain level. Through the comparison of different data sets (mitochondrial dysfunctions vs. Fe- and/or S-responsive genes), nutrient-responsive genes have been identified as potential candidates of hypothetical retrograde signaling pathways (PYE, BTS, FRO3; OPT3, SAM1 for Fe homeostasis and SULTR1;2 SULTR3;4, SULTR4;2, LSU1, BGLU28, SDI1 for S homeostasis). Mitochondrial impairments occur in long term of Fe and S starvations; therefore, such retrograde signaling would occur in a second phase of the regulation of nuclear gene expression, whereas the first phase (gray arrows) involved a more direct nutrient-sensing mechanisms.

It has been suggested that Fe and S deficiencies might trigger retrograde signaling pathways to regulate the expression of genes. However, no clear evidence demonstrating such hypothesis has been reported so far. Considering that both Fe and S deficiencies affect mitochondrial respiration, it can be hypothesized that the impaired respiratory chain might be at the origin of putative retrograde signals under such nutritional deficiencies. In agreement with such a hypothesis, we observed that the expression of several Fe- and S-responsive genes was affected in plants with an induced mitochondrial dysfunction, as reported in the study by Schwarzländer et al. (2012). Interestingly, these genes exhibited a similar regulation (up or down) under Fe or S deficiencies and respiratory chain impairments. These observations underline the possible role of the respiratory chain impairment under Fe and S deficiencies in the regulation of some Fe and S-responsive genes. Here, we hypothesize that Fe and S deficiencies, by triggering mitochondrial impairments, promote the generation of specific signal(s) targeted from this organelle to the nucleus, leading to the up-regulation of nuclear-encoded genes involved in the establishment of Fe and S homeostasis, respectively (Figure 1). However, mitochondria might be considered only as one of the players in the complex nutrient-sensing and signaling mechanisms, as other more direct regulations occur (Figure 1). Indeed, it has been proposed that direct binding of Fe to transcriptional regulators would be the primary Fe-sensing event in plant cells (Kobayashi and Nishizawa, 2014, 2015). Putative retrograde signaling pathways might, therefore, occur in a second time of nutrient sensing. Considering that mitochondrial impairments should occur on the long-term in response to Fe or S starvation, we suggest that mitochondria might come into the regulation of nutrient-responsive genes at a later stage. Plant responses to nutrient deficiencies, such as Fe or S, are more complex than the simple activation of nutrient uptake/translocation mechanisms. Indeed, a complex metabolic reprogramming occurs in the cell in order to adapt itself to the low nutrient availability, as in the case of Fe (Vigani, 2012).

Interestingly, among the Fe-responsive genes reported by Stein and Waters (2012), 45% of them were similarly regulated under Fe deficiency and mitochondrial dysfunctions. Furthermore, the expression of 45% of the PYE-regulated genes was affected by mitochondrial dysfunctions. Whereas among the S-responsive genes considered (Maruyama-Nakashita et al., 2006), 38% of them were similarly regulated under S deficiency and mitochondrial dysfunction. Complex I impairments seem to induce a differential expression of the majority of Fe- and S-responsive genes (Tables 1–3). It is well known that complex I deficiencies dramatically impact on cellular physiology, and numerous diseases have been linked with the impairment of complex I (Kühn et al., 2015). In humans, complex I is the main origin of diseases resulting from mitochondrial dysfunction (Nouws et al., 2012). However, in plants, the absence of complex I does not cause premature death as it occurs in humans (Kühn et al., 2015 and reference therein). Indeed, in plants, bypasses do exist for complex I (i.e., ND_ex_), allowing even more metabolic flexibility than in human mitochondria. Recently, a central role of ND_ex_ in the regulation of cellular metabolism has been suggested. Suppression of the external mitochondrial NADPH dehydrogenase NDB1 affects global gene expression in *Arabidopsis* plants (Wallström et al., 2014), thereby suggesting that changes in nicotinamide redox level can selectively affect particular process of the cell. In fact, the existence of a NAD(P)H signaling process in plants has been suggested (Noctor, 2006). Under both Fe and S deficiencies, the increased activity of ND_ex_ along with the decreased activity of complex I might indicate that both nutrient deficiencies affect the nicotinamide redox level of the cell. Therefore, a possible NAD(P)H signaling pathway might be involved in the regulation of such nutrient-responsive genes. In this case, the metabolic status of mitochondria might produce non-specific signals. Therefore, the regulation of nutrient-responsive genes might result from integrated signaling pathways, involving specific and non-specific signals.

However, it cannot be ruled out that the above-reported overlap in the gene expression response to mitochondria dysfunctions or to Fe or S deficiencies could be simply be random and due to transcriptional noise, rather than indicative of a specific induction of nutrient starvation responses. Indeed, it is important to keep in mind that transcription is intrinsically stochastic and a small overlap may or may not be of physiological significance.

Furthermore, the transcriptomic data sets used here were obtained from different plant materials: cell culture, plant tissues, and at different plant developmental stages. It would be, therefore, important to investigate the possible role of mitochondria in the regulation of gene expression, specifically by differentiating cell culture from plant tissues and by differentiating root from shoot tissues of plant sampled at a given developmental stage. The cross-talk between mitochondria and plastids in roots should differ from that one between mitochondria and chloroplasts, and thereby the effect of respiratory chain impairment might be integrated in different signaling pathways in roots and leaves.

In order to clarify these points, it will be important to elucidate whether the impairment of the respiratory chain occurring under Fe and S deficiencies might affect the expression of some nutrient-regulated genes or whether it should be considered simply as a non-specific effect of the nutrient starvation. As the putative signals generated by impaired mitochondria might be numerous, the characterization of the retrograde pathway occurring under nutritional disorder could be complex. However, in order to elucidate the possible impact of the impaired respiratory chain on the expression of crucial Fe- and S-responsive genes, it would be helpful to evaluate the impact of respiratory chain inhibitors on the expression of Fe- and S-responsive genes in wild type and mutant plants that are defective in expression of important transcription factors (PYE/BTS, for Fe and SLIM, for S).

## Author contributions

GV conceived the idea and wrote the draft of the manuscript. JFB critically reviewed for important intelectual content the manuscript.

### Conflict of interest statement

The authors declare that the research was conducted in the absence of any commercial or financial relationships that could be construed as a potential conflict of interest.
